# An in vitro investigation of telocytes-educated macrophages: morphology, heterocellular junctions, apoptosis and invasion analysis

**DOI:** 10.1186/s12967-018-1457-z

**Published:** 2018-04-03

**Authors:** Xiao-Juan Jiang, Dragos Cretoiu, Zong-Ji Shen, Xiao-Jun Yang

**Affiliations:** 1 Department of Obstetrics and Gynecology, The First Affiliated Hospital of Soochow University, 188 Shizi Road, Suzhou, 215006 Jiangsu Province People’s Republic of China; 2 Division of Cellular and Molecular Biology and Histology, Carol Davila University of Medicine and Pharmacy, Bucharest, Romania; 3 Materno-Fetal Assistance Excellence Unit, Alessandrescu-Rusescu National Institute of Mother and Child Health, Bucharest, Romania

**Keywords:** Telocytes (TCs), Interstitial cells, Peritoneal macrophages (pMACs), Macrophage activation, Mitochondrial membrane potential (ΔΨm), Heterocellular junctions, Apoptosis, Endometriosis (EMs), Immunoregulation

## Abstract

**Background:**

Telocytes (TCs), a recently discovered novel type of interstitial cells, were also found in a wide variety of human and mammalian reproductive organs/tissues, including uterus, oviduct and placenta. Previously, we demonstrated that TCs-conditioned media was capable of activating peritoneal macrophages (pMACs) through paracrine effects. This study investigates the hypothesis that direct interaction of TCs with pMACs will also play a significant role in immunoregulation of pMACs.

**Methods:**

TCs and pMACs were derived from the uterus and intraperitoneal cavity of female BALB/c mice, respectively. TCs were identified by immunofluorescence and then co-cultured directly with pMACs for 24 h without added cytokines, to observe the in vitro biological behavior of pMACs. We used histochemical staining to study morphology and mitochondrial metabolism of pMACs, scanning electron microscopy to study heterocellular junctions, flow cytometry to investigate mitochondrial membrane potential (ΔΨm) and apoptosis, and transwell chambers to study invasion ability. Student-t test was used accordingly.

**Results:**

Presently, TCs with typical structure and immunophenotype of double CD-34-positive/vimentin-positive were successfully isolated. pMACs co-cultured with TCs showed obviously morphological activation, with enhanced energy metabolism (*P *< 0.05). Meanwhile, direct physical cell-to-cell interaction promoted the development of heterocellular junctions between TCs and pMACs. Furthermore, TCs treatment markedly reduced the depletion of ΔΨm in co-cultured pMACs (all *P *< 0.05), and inhibited their apoptosis (*P *< 0.05). Functionally, pMACs co-cultured with TCs showed enhanced invasion ability (*P *< 0.05).

**Conclusions:**

Direct physical cell-to-cell interaction promoted the development of heterocellular junctions between TCs and pMACs, presumably responsible for the observed novel efficient way of pMACs activation via mitochondrial signaling pathway. TCs-educated pMACs might be a promising way to restore the defective immunosurveillance in endometriosis (EMs), led to the enhanced treatment efficacy of EMs in a simple and clinically feasible fashion.

## Background

Telocytes (TCs), a special type of interstitial cells, firstly identified by Popescu’s group, were also found in female reproductive organs, including the uterus, fallopian tubes, placenta [1–11]. TCs were characterized with a small cellular body and very long prolongations named telopodes (Tps), which contain an alternation of dilations (podom) and thin segments (podomer) [12–21]. TCs develop heterocellular junctions with various adjacent cells through Tps and affect their activities. The exact functions of TCs were supposed to maintain tissue homeostasis, intercellular communication and tissue repair/remodeling [4, 5, 11, 22–37], even though these remain to be proved.

In our previous animal studies, with the aid of transmission electron microscopy, TCs was found to have specific ultrastructural features and was essential for the maintenance of the structural and functional integrity of oviduct tissue. Meanwhile, by their Tps, TCs connected to various activated immunocytes (mononuclear cells, mast cells, eosinophils and neutrophils) via heterocellular junctions both in normal and disease-affected oviduct tissues [6, 7]. Furthermore, when indirectly co-cultured with mice peritoneal macrophages (pMACs) in vitro, we found that TC-conditioned media (TCM) can activate pMACs, trigger and maintain an immune response, likely through indirect paracrine effects [38]. Interestingly, many other studies also reported that TCs were capable of developing heterocellular junctions with a plethora of immunocytes (eosinophils, macrophages, plasma cells etc.) in different tissues and organs [2, 29, 30, 39–43]. Thus presumably suggested potential immunoregulation roles of TCs in local immuno-inflammatory processes through direct junctions or paracrine effects, either repression or activation their responses.

On the other hand, macrophages are the most important and widely distributed immunocytes, and are also a key component of innate immunity located in the peritoneal/pelvic cavity. They can remove various benign and malignant cell components from the female reproductive tract, to prevent immune-related gynecologic diseases, such as endometriosis (EMs), infertility, pre-cancerous and cancerous diseases. Defective immunosurveillance of female pelvis, especially the immune dysfunction of pMACs, such as pathological accumulation and/or functional alterations of pMACs, probably changes the survival, growth and spread of autologous endometrial cell debris contained in the retrograded menstrual blood, thus contributing to the pathogenesis of EMs, and immune-mediated reproductive problems [44–50].

To further reveal unique immunological properties of TCs, this study investigates the hypothesis that direct interaction of TCs with pMACs will also play a significant role in immunoregulation of pMACs. Currently, pMACs were directly co-cultured with TCs and further investigated their biological behavior in vitro, including morphology study by histochemical staining, heterocellular junctions by scanning electron microscopy (SEM), mitochondrial membrane potential (ΔΨm) and apoptosis assay by flow cytometry, invasion ability by transwell chambers assay. Such knowledge will be helpful to add more information on local immunoregulation/immunosurveillance roles of TCs and potential functional consequence on immune-related EMs, encourage the development of novel diagnosis and treatments for diseases with proved immune etiology.

## Methods

### Animals

Female BALB/c mice (20–25 g) aged 8–10 weeks, were obtained from the Animal Research Center of Soochow University, Suzhou, China. Mice were housed in a specific pathogen-free environment with full access to water and food before experiments. All experimental protocols were approved by The Institutional Animal Care and Use Committee at Soochow University.

### Isolation and primary culture of TCs

Uterine tissues were harvested from the mice killed with excessive sodium pentobarbital (50 mg/kg; Fuyang Pharmaceutical Factory, Fuyang, China) under sterile conditions. Then tissues blocks were washed three times with phosphate buffered saline (PBS) (Sigma-Aldrich, St. Louis, MO, USA), cut into 1 mm^3^ fragments and incubated in DMEM/F12 (Gibco, New York, USA) supplemented with 0.1% collagenase type II (Sigma-Aldrich) for enzymatic dissociation at 37 °C, combined with mechanical dissociation with mild agitation through a Pasteur pipette every 15 min. After 90 min, 10% FBS (Gibco) was added gradually to quench the enzymatic reaction. Collected cells were then centrifuged (302*g* for 10 min) and re-suspended in DMEM/F12 with 10% FBS, seeded in plastic 25-cm^2^ culture flasks (Corning Inc., Corning, NY) and incubated in a humidified CO_2_ incubator (5% CO_2_ with 95% air) at 37 °C for 2 h to prepare for TCs monolayer attachment. Then the culture medium was replaced with a complete medium which was changed every 48 h thereafter. After 3–4 days of primary cell culture, uterine TCs were transferred to 6-well culture plates containing 2.5 ml of serum-free DMEM/F12 to reach a final concentration of 5 × 10^4^ dissociated cells/well.

### Double-labelled immunofluorescence cytochemistry

Fresh TCs was harvested and smeared on slides, then fixed in 4% paraformaldehyde for 20 min, washed with PBS three times and permeabilization with 0.5% Triton X-100 for 15 min, then blocked in 3% bovine serum albumin (BSA) for 1 h. After that, cells were incubated with primary antibodies: rat anti-vimentin (1:100; cat. no. 5741, Cell Signaling, USA) and rabbit anti-CD34 (1:200; cat. no. ab81289; Abcam) overnight at 4 °C. After washed three times with PBS, cells were exposed for 1 h at 37 °C to goat anti-rat lgG-FITC (1:100; cat. no. sc-2011, Santa Cruz, USA) and goat anti-rabbit IgG-CY3 (1:400; cat. no. ab97075; Abcam), then counterstained with DAPI (1:50; cat. no. C1002, Beyotime, Shanghai, China). Finally, cells slides were mounted with antifade medium (1:1000; cat. no. p0126; Beyotime, Shanghai, China) and photographed under a fluorescent inverted microscope (Leica DMI4000 B, Germany).

### Isolation of pMACs and direct co-culture with TCs

Briefly, BALB/c mice received an intraperitoneal injection of 2 ml of 3% thioglycollate (Sigma-Aldrich) for pMACs enrollment. Then 3 days later, mice were killed with an overdose of sodium pentobarbital via a subcutaneous injection, then pMACs were harvest from the intraperitoneal cavity and isolated, counted microscopically using trypan blue dye. Then pMACs (5 × 10^5^ dissociated cells/well) was inoculated into 6-well plates to directly co-cultured with TCs (5 × 10^4^ dissociated cells/well) at a ratio of about 10:1. The co-cultured TCs and pMACs were examined for morphologic alterations at the time points of 0, 12 and 24 h, respectively and imaged under light microscope (Leica, Heidelberg, Germany).

### Crystal violet staining of co-culture system for morphology observation

After direct co-cultured for 24 h, TCs and pMACs were harvested and smeared on slides, washed three times with PBS, fixed in 4% paraformaldehyde for 20 min and stained with crystal violet solution (0.01 mg/ml, Beyotime, Shanghai, China) at room temperature for 10 min, then washed with PBS again and imaged under light microscope.

### Mitochondrial labelling

Mitochondria labelling was used to detect energy metabolism alterations of pMACs and TCs in the direct co-cultured system. At the time point of 0 and 24 h, the co-cultured cells (same cell ratio as above-mentioned) were incubated in phenol red-free DMEM, with 100 nmol/l MitoTracker Green (Beyotime, Shanghai, China) for 30 min, in a humidified atmosphere (5% CO_2_ at 37 °C). Then, the fluorescence intensity of the co-cultured system was detected three times in each group under fluorescence microscopy (450–490 nm excitation light, 520 nm barrier filter; Leica DMI4000 B, Germany). Meanwhile, same amount of pMACs without TCs served as control conditions. Mean fluorescence intensity (MFI) was semi-quantitatively analyzed by Image-Pro Plus 6.0 software (Media Cybernetics, Silver Spring, USA), for 0 and 24 h, respectively [51].

### Scanning electron microscopy of the direct co-cultured samples

At the time point of 24 h, the freshly direct co-cultured samples (containing pMACs 5 × 10^5^ and TCs 5 × 10^4^) were harvested, smeared on slides, washed three times with PBS to remove cell debris (5 min for each change), then fixed as the following: 3% buffered glutaraldehyde (pH 7.2) for 30 min at room temperature followed by PBS washing; dehydrated through graded ethanol (35, 50, 75, 90, 100%) (5 min for each change); then pre-processed with vacuum deposition of gold plating. TCs and pMACs were observed and imaged under HITACHI S-450 scanning electron microscope (Tokyo, Japan).

### Annexin V-FITC/PI double staining

Apoptosis of pMACs was determined by annexin V-FITC/PI staining. pMACs in the co-cultured and cultured-alone groups were exposed to the following three conditions for induction of apoptosis, respectively: serum-free DMEM/F12 for control conditions, irradiated at 15 W ultraviolet lamp 10 min for positive control (UV, a physical induction of apoptosis stimulus), serum-free DMEM/F12 with 80 μg/ml dexamethasone for positive control (DXM, Sigma-Aldrich, a chemical induction of apoptosis stimulus). After 24 h of apoptotic induction, pMACs in both groups were added with 5 μl of FITC-conjugated Annexin V (Annexin V-FITC) and 5 μl of propidium iodide (PI) (both from Dojindo Laboratories, Kumamoto, Japan) and incubated for 10 min at room temperature in the dark according to the manufacturer’s protocol, then 400 μl binding buffer was added to each tube. Annexin V-FITC/PI-stained cells were measured by flow cytometry within 1 h (excitation wavelength 488 nm, emission wavelength 530 nm; Becton–Dickinson; Becton–Dickinson, San Jose, CA) and the acquired data were analyzed by CellQuest software. The population of Annexin +/PI + cells was defined as late apoptosis/secondary necrosis, while annexin +/PI − cells was defined as early apoptosis [52].

### Mitochondrial membrane potential (ΔΨm) assay

The ΔΨm was measured by using mitochondria-specific cationic dye (JC-1, Sigma, USA) and Rhodamine-123 dye (Rh–123, Dojindo Laboratories, Kumamoto, Japan), respectively. Briefly, pMACs (1 × 10^6^ cells/well) were inoculated into TCs (1 × 10^5^ cells/well) in 6-well plates, with the same number of pMACs cultured alone in 6-well plates for control conditions. The non-adherent cells were removed after incubation at 37 °C for 4 h to allow pMACs to adhere to the surface of the plastic culture plates in both groups. Then, pMACs in both groups were treated with above-mentioned two stimuli (UV and DXM) and control conditions. After 24 h of apoptotic induction, pMACs in both groups were collected by centrifugation at 302*g* for 10 min.

First, JC-1 was employed to analysis mitochondrial depolarization in pMACs. After induction of apoptosis, pMACs in both samples (1 × 10^6^ cells/well) were incubated in DMEM/F12 with 2.5 µg/ml JC-1 in the dark at 37 °C for 30 min. Then determined by flow cytometry (Becton–Dickinson, San Jose, CA) immediately. JC-1 enters, aggregates in mitochondria of non-apoptotic cells and turns red, but gathered in the cytosol of apoptotic cells and turns green. The ratio of green to red fluorescence intensities reflects tendency of negative changes in ∆Ψm.

Second, Rhodamine fluorescence was employed to analyze mitochondrial membrane polarization of pMACs. Similarly, after induction of apoptosis, pMACs in both samples (1 × 10^6^ cells/well) were incubated with 1 mM Rh-123 at 37 °C for 0.5 h. Then washed with cold PBS three times and immediately analysis by FL1 channel of flow cytometry for the detection of fluorescence intensity, which showed a positive tendency to ΔΨm.

### Cell invasion assays

In order to observe invasion ability of pMACs in response to TCs exposure, Transwell chambers assay based on Matrigel invasion was performed (24-well insert, pore size 8 µm; BD Biosciences, Bedford, MA, USA). The upper chambers were filled with serum-free DMEM/F12 and mixed with Matrigel (BD Biosciences, USA) (1:7 dilution) to solidify at room temperature for 3 h. pMACs was seeded onto the top chamber in 400 µl serum-free medium to reach a concentration of 5 × 10^4^ cells/ml. TCs was harvested with trypsin, washed, resuspended in serum-free DMEM/F12, and added to the lower chamber to reach a concentration of 4 × 10^3^ cells/ml, which contained 10% FBS as chemoattractant. After incubated at 37 °C, 5% CO_2_ for 24 h, the chambers were fixed and stained with crystal violet solution (0.01 mg/ml, Beyotime, Shanghai, China). The average number of pMACs invasion on the lower surface of membrane was counted in five individual random high-powered fields (400×) for each membrane and images were captured under phase-contrast microscope. Assays were performed in triplicate for each treatment group.

### Statistical analysis

The data were expressed as mean ± standard deviation (SD), and statistical analysis was performed by using SPSS software (version 13; SPSS Inc., Chicago, IL, USA). Student’s *t* test was applied for two independent samples. A difference with *P *< 0.05 was considered statistically significant.

## Results

### Isolation and identification of TCs

**Fig. 1:**
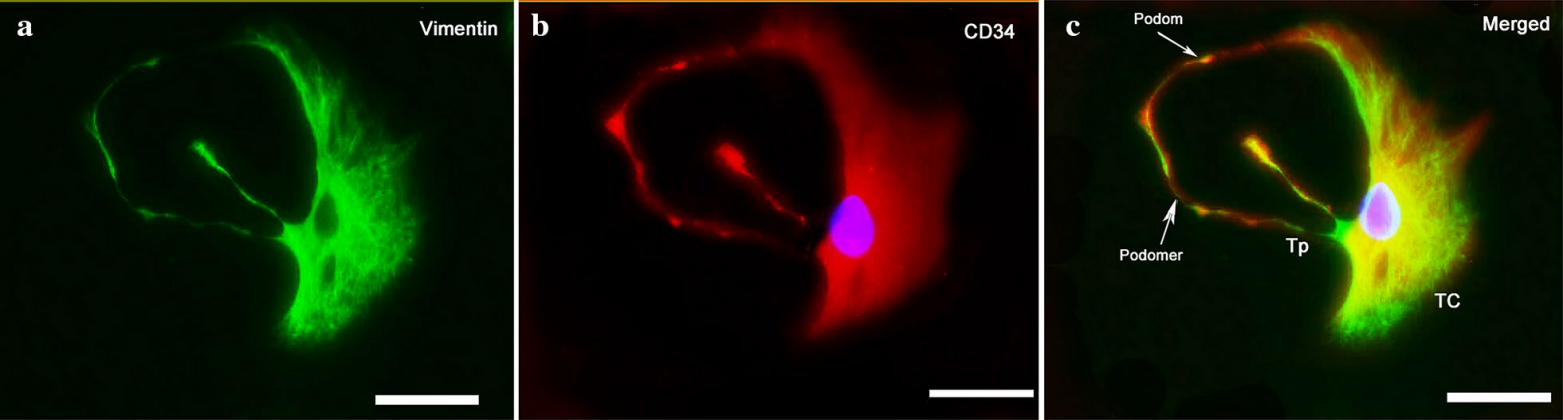
Representative double-labeled immunofluorescence identification of TCs. Images of c-kit negative staining were not shown; Scale bar = 50 μm. **a** Positive FITC labelling for Vimentin (green). **b** Positive CY3 labelling for CD34 (red). Nuclei were counterstained with DAPI (blue). **c** In the merged image, Vimentin and CD34 were overlapped, both in the cellular body and full length of Tp, with characteristic podomer (thin segments) and podom (thick segments) arrayed alternatively along Tp

Typical uterine TCs can be identified by characteristic structures and immunophenotype after 3 or 4 days of primary cell culture. TCs demonstrated with positive vimentin fluorescence (green)/CD34 fluorescence (red) in its cellular body and the whole length of Tps, which was characterized with alternations of thin (podomer) and thick segments (podom) (Fig. 1a, b). And in the merged image, both kinds of immunofluorescence overlapped each other and manifested a yellow color, with intact DAPI nuclei (blue) (Fig. 1c). Thus, we confirmed that the special immunophenotype for TCs was CD-34-positive/vimentin-positive/c-kit-negative.

### Morphological alterations of TCs and pMACs

**Fig. 2:**
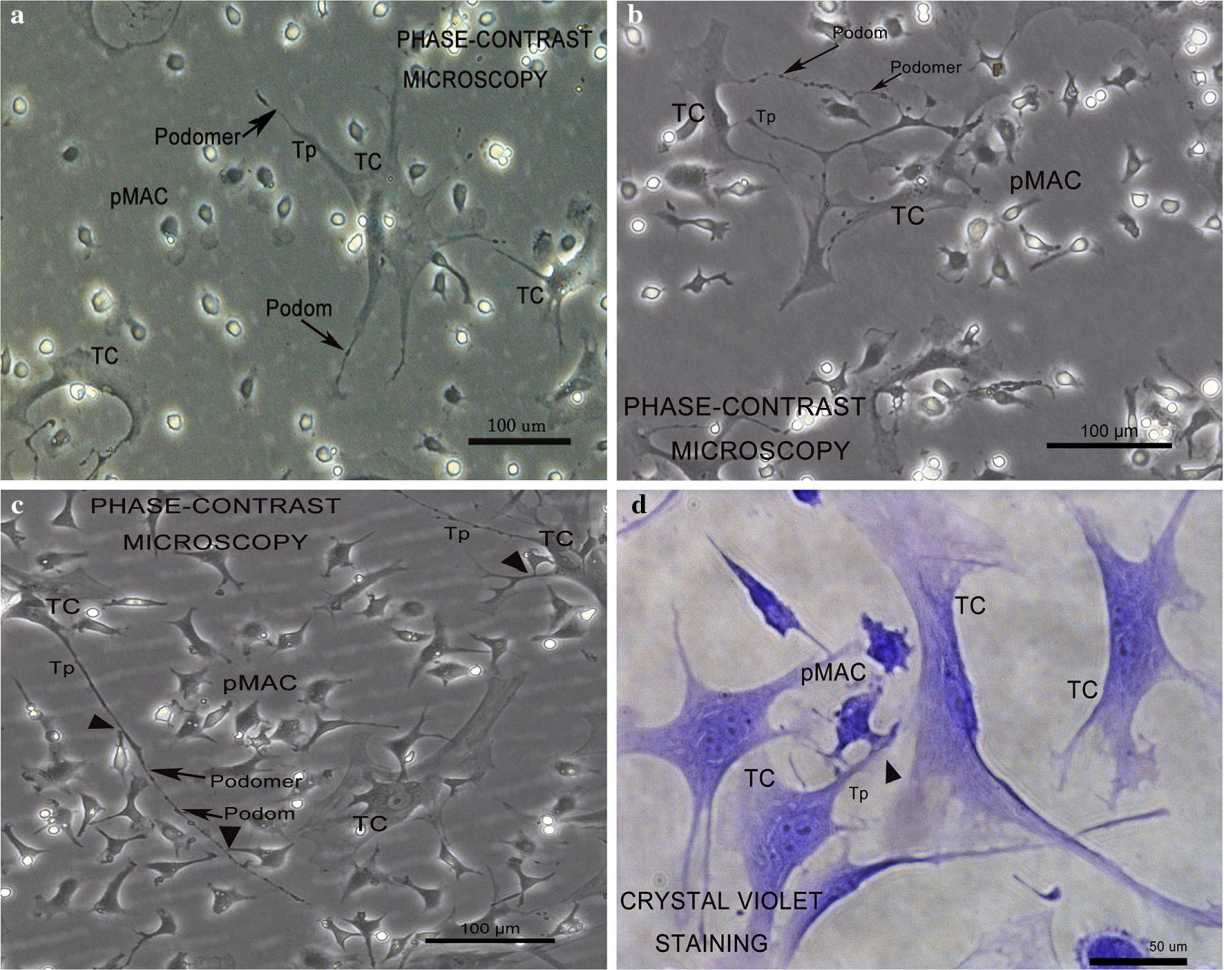
Real-time dynamic observation of primary directly co-cultured TCs and pMACs at 12 h intervals under phase-contrast microscopy; mice uterus. TCs have small oval cell bodies and long Tp branched from the cell body, appearing as an alteration of podom and podomer. **a** At 0 h, pMACs showed its normal regular round shape and no signs of activation. **b** At 12 h, pMACs showed moderate activation with irregular polyhedron shape and pseudopodia, but without any intercellular contacts to TCs. **c** At 24 h, pMACs exhibited intensive morphological changes and continuous activation, with obvious polyhedron, abundant pseudopodia; more importantly, direct heterocellular junctions can be observed between the activated pMACs and TCs (black arrowhead). **d** At 24 h, vital staining with crystal violet demonstrated heterocellular junction between the pseudopodia of activated pMACs and TCs (black arrowhead)

In a co-cultured system, under phase contrast microscopy, TCs showed typical slender piriform-shaped cellular body, and one or more extremely long, thin Tps, with characteristic podomer and podom (Fig. 2). Nevertheless, no special morphologic changes occurred for TCs in time-lapse observation within 24 h.

On the other hand, pMACs changed gradually and indicated significant activation during the whole process (Fig. 2a–c). First, at the time point of 0 h, pMACs kept its original regular round shape when seeded into TCs, indicated no changes in activation (Fig. 2a). Then, at 12 h, some pMACs changed into polyhedron shapes, simultaneously with pseudopodia extending from the cell membrane, indicated moderate activation of immune response (Fig. 2b). Finally, at 24 h of direct co-culture, pMACs was in an intensified activation/immune response, which exhibited considerable morphological differences, including more complex polyhedron shapes and abound pseudopodia, accompanied by direct heterocellular junctions between pMACs and TCs (Fig. 2c). On the other hand, at 24 h, crystal violet staining also showed heterocellular junctions between the pseudopodia of the activated pMACs and Tps (Fig. 2d).

### Energy metabolism status of pMACs

**Fig. 3:**
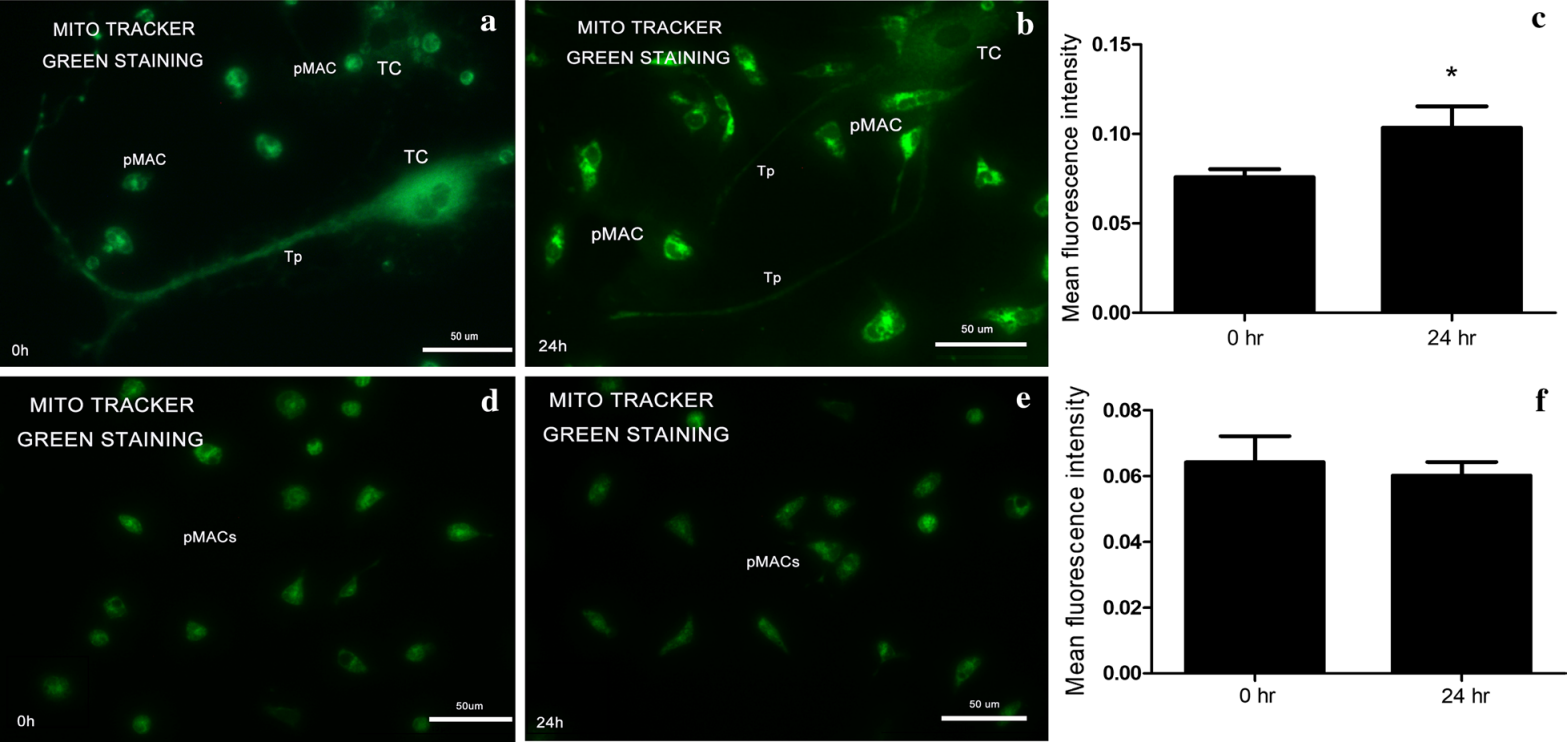
Fluorescence microscopy of Mito Tracker green staining. Error bars = SD. The MFI of each group was repeatedly detected three times under the same conditions. **a**–**c** The observed fluorescent intensity for TCs and pMACs in the co-cultured system at two time points. MFI at 24 h was significantly higher than that of 0 h (*P < 0.05), indicating active energy metabolism of pMACs in the co-cultured system. **d**–**f** No significant difference for background MFI of cultured-alone pMACs was found in two time points

Energy metabolism of pMACs and TCs was detected by mitochondrial labelling and semi-quantitative analysis of MFI for each group at 0 and 24 h, respectively. As indicated in Fig. 3a–c, MFI value of mitochondrial increased significantly at 24 h when compared to that of 0 h in the direct co-cultured system (0.1034230 ± 0.012060 at 24 h versus. 0.0758212 ± 0.004438 at 0 h; *P *< 0.05). Meanwhile, in Fig. 3d–f, no significant difference for background MFI of cultured-alone pMACs was found in two time points (0.0600895 ± 0.001697 at 24 h versus. 0.0641546 ± 0.003243 at 0 h; *P *> 0.1).

Based on the observed phenomenon that: (a), no special morphologic changes occurred for TCs, while obvious activation of pMACs at 24 h; (b), the ratio of pMACs and TCs was 10:1 in the co-cultured system; (c), more importantly, no significant difference of the background MFI for cultured-alone pMACs in two time points, therefore, here we can approximately take the total MFI changes as the value of pMACs. Thus, the above results indicated active energy metabolism of pMACs at 24 h in the co-cultured system.

### Heterocellular junctions between TC and pMACs observed by SEM

**Fig. 4:**
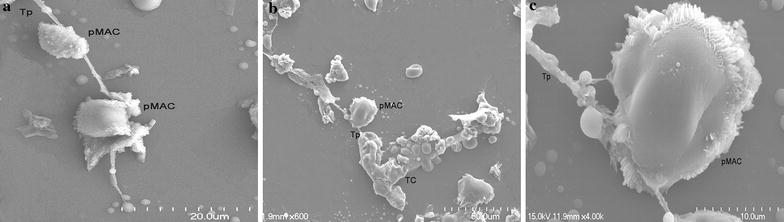
**a**–**c** Scanning electron microscopy of the direct co-cultured system at 24 h. TCs displayed oval cell body with long TP. The activated pMACs with irregular shape, abundant fluff-like structures on the surface of the cell membrane was observed. The complex direct heterocellular junction between the activated pMACs and Tp was presumably a certain type of immunological synapse (IS)

After co-cultured for 24 h, under SEM observation, TCs can be identified with a small cellular body and characteristic moniliform branched TP. The activated pMACs with irregular shape can be observed with an abundant fluff-like structure on the surface of cell membrane. Most importantly, SEM reveals direct heterocellular junctions between TP and activated pMACs (Fig. 4a–c).

### Apoptosis of pMACs

**Fig. 5:**
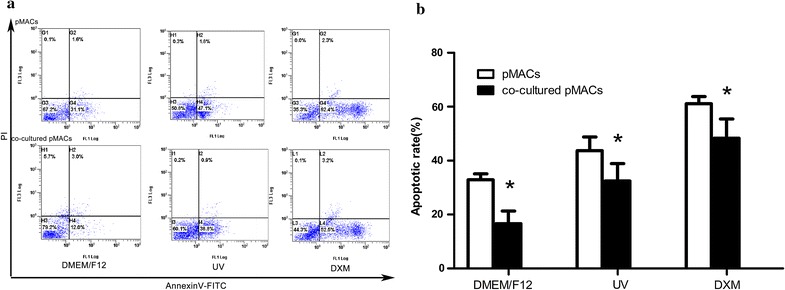
Flow cytometry analysis of apoptosis of pMACs (cultured-alone, co-cultured with TCs) under three conditions (DMEM/F12 for control conditions, UV irradiation and DXM for positive control). **a** Annexin V-FITC/PI double staining method was used to quantitatively analysis the percentage of apoptotic cells. **b** The considerably lower percentage of apoptosis was detected for the co-cultured pMACs, compared with that of cultured-alone pMACs in all of three conditions (all *P < 0.05 versus cultured-alone pMACs). Error bars = SD. The mean and SD were calculated from three independent experiments

Apoptosis of pMACs in both groups, which were treated with above-mentioned two stimuli (UV and DXM) and control conditions, were examined by Annexin V-FITC/PI double staining and flow cytometry. The apoptotic proportion was defined as the percentage of cells with the fluorescence of Annexin +/PI − and Annexin +/PI + . As shown in Fig. 5a, b, under the control conditions, the rate of apoptosis of the co-cultured pMACs (16.63 ± 4.669%) was significantly lower than cultured-alone pMACs (32.9 ± 2.207%) (*P *< 0.05). When exposure to UV, the co-cultured pMACs (32.43 ± 6.452%) was significantly lower than cultured-alone pMACs (43.7 ± 5.048%) (*P *< 0.05); and in DXM group, the co-cultured pMACs (48.3 ± 7.163%) was significantly lower than cultured-alone pMACs (61.07 ± 2.684%) (*P *< 0.05). Thus, under three conditions, the considerably low percentage of apoptotic pMACs were detected in the co-cultured system compared with those of cultured-alone pMACs.

### Mitochondrial membrane potential (∆Ψm) changes

**Fig. 6:**
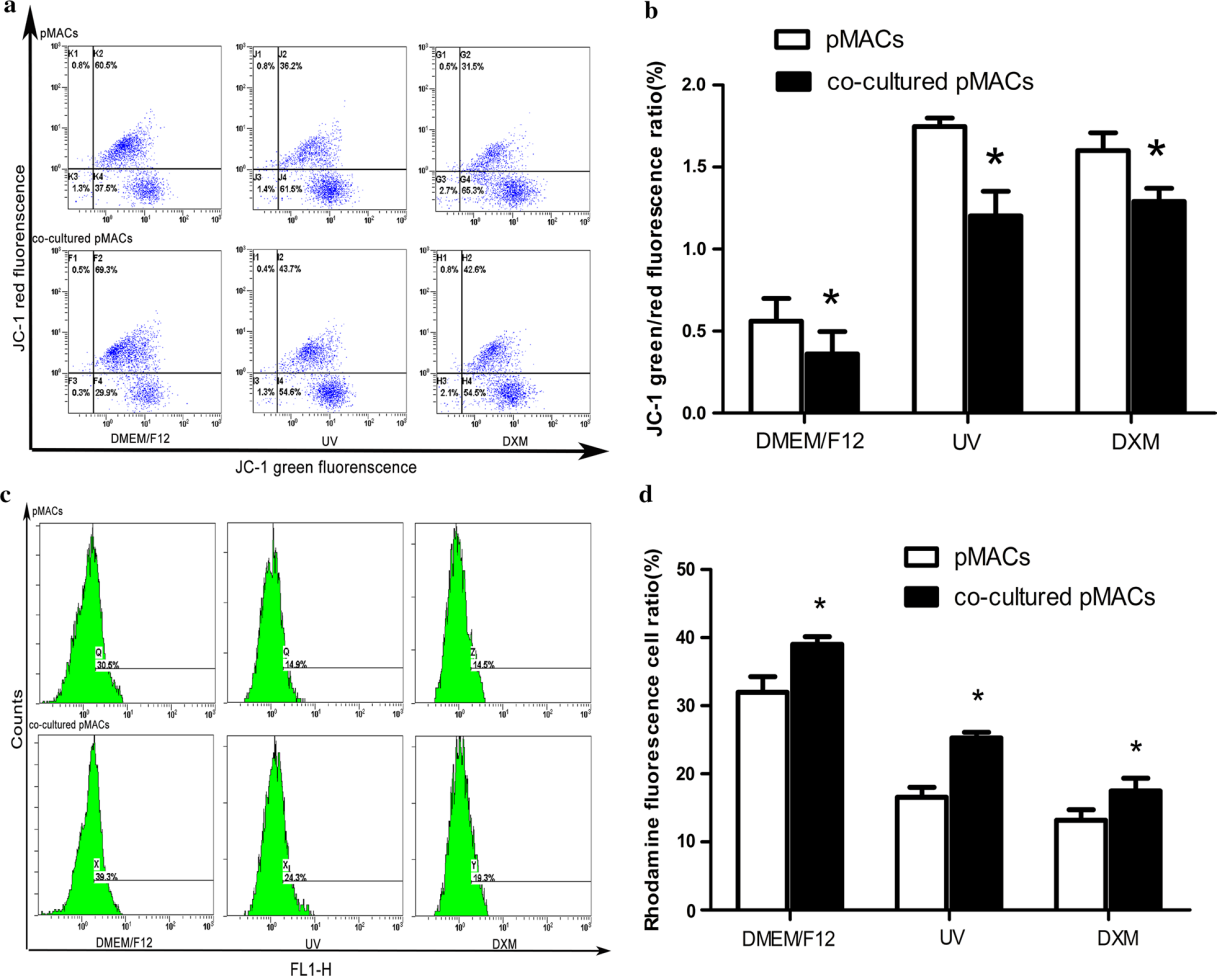
Changes of ∆Ψm in pMACs (cultured-alone, co-cultured with TCs) were evaluated by flow cytometry analysis of JC-1 and Rhodamine fluorescence, respectively, under three conditions (DMEM/F12 for control conditions, UV irradiation and DXM for positive control). Results showed that the depletion of ∆Ψm in pMACs can be reduced by TCs, further prevent their apoptosis. Error bars = SD. The mean and SD were calculated from three independent experiments. **a** For JC-1, value of ∆Ψm was represented as the green/red fluorescence ratio by flow cytometry. **b** For both groups of pMACs, higher values of ∆Ψm were detected in cultured-alone pMACs compared with that of co-cultured pMACs, in all of three conditions (all *P < 0.05 versus cultured-alone pMACs). **c** For Rhodamine, value of ∆Ψm was represented as Rhodamine fluorescence intensity by flow cytometry. **d** For both groups of pMACs, higher values of ∆Ψm were detected in the co-cultured pMACs compared with that of cultured-alone pMACs, in all of three conditions (all *P < 0.05 versus cultured-alone pMACs)

In order to investigate potential pathway of apoptosis of pMACs, which were treated with above-mentioned two stimuli (UV and DXM) and control conditions, ∆Ψm was further studied by JC-1 and Rhodamine fluorescence, respectively. As indicated in Fig. 6a–d, results of these changes showed that, when directly co-cultured with TCs in vitro, the loss of ∆Ψm of pMACs was reduced and apoptosis of pMACs was prevented via a mitochondrial pathway.

First, for JC-1 analysis, as shown in Fig. 6a, b, the results of green/red fluorescence ratio of the co-cultured pMACs were (0.3613 ± 0.1357), (1.203 ± 0.1499), (1.291 ± 0.08043) for DMEM/F12, UV irradiation and DXM, respectively, all were significantly lower than that of cultured-alone pMACs (0.5608 ± 0.1377), (1.748 ± 0.05079), (1.601 ± 0.1082) (all *P *< 0.05). Such results showed that loss of ∆Ψm in pMACs was markedly decreased by TCs.

On the other hand, similar results were observed in Rhodamine fluorescence assay. As shown in Fig. 6c, d, the Rhodamine fluorescence cell ratio of the co-cultured pMACs were (39.03 ± 1.124%), (25.27 ± 0.8387%), (17.47 ± 1.904%) for DMEM/F12, UV irradiation and DXM, respectively, all were significantly higher than that of cultured-alone groups (31.97 ± 2.285%), (16.57 ± 1.447%), (13.20 ± 1.51%) (all *P *< 0.05). Such results showed that ∆Ψm in pMACs was markedly increased by TCs.

### Invasion ability of pMACs

**Fig. 7:**
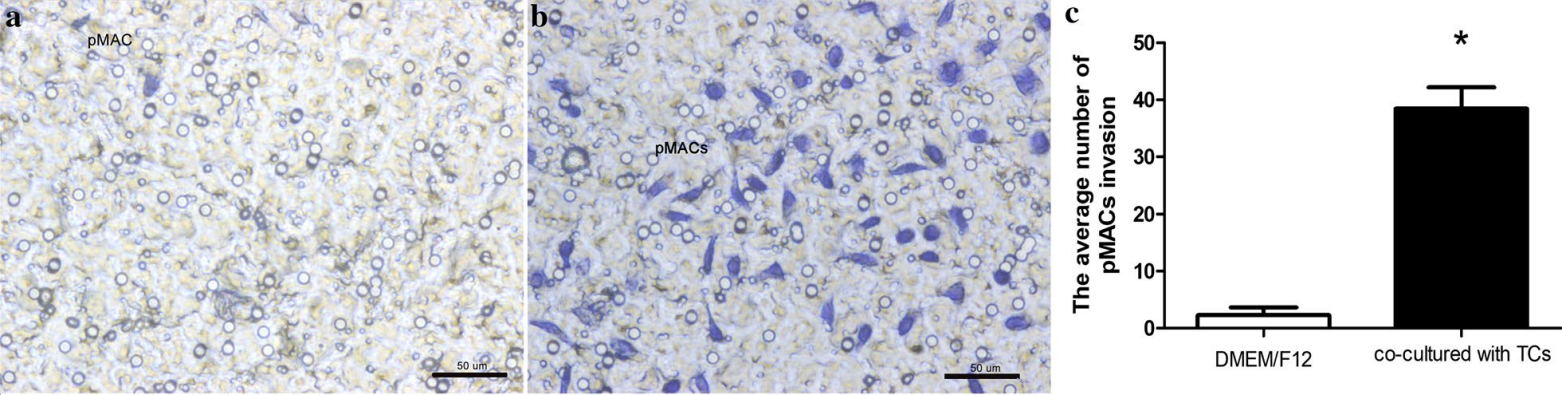
Invasion assay of pMACs (blue dye cells) after 24 h by Transwell chambers assay. **a** When cultured alone, very few pMACs invasion can be observed on the lower surface of the membrane. **b** When co-cultured with TCs, cellular invasion of activated pMACs was significantly higher than that of cultured-alone pMACs. **c** The average number of pMACs invasion significantly increased. (*P < 0.05 versus DMEM/F12). Error bars = SD. The mean and SD were calculated from three independent experiments

Transwell chambers was used to study invasion ability of pMACs. Results showed that after co-cultured for 24 h, the average number of pMACs invasion in co-cultured group (38.43 ± 9.981) increased significantly than in the cultured-alone groups (2.286 ± 3.592) (Fig. 7a–c) (*P *< 0.05). Moreover, the pMACs were obviously activated with polyhedron shapes and typical pseudopodia (Fig. 7b).

## Discussion

TCs has been identified to exist in female reproductive organs, with its exact functions still being exploring [1–11]. However, it has been proved that their unique structures allow them to form heterocellular junctions with various immunocytes, both in normal and diseased-affected tissues [2, 29, 30, 39–43, 53, 54]. Previously, we reported that pMACs can be activated by TCM via indirect autocrine/paracrine mechanisms, leading to an increased production of pMACs-derived cytokines. This can trigger and maintain an immune response, potentially playing an important role in reproductive physiology [38]. To further investigate their direct cell-to-cell interactions with pMACs and explore its unique immunological properties, in the current study, uterine TCs was directly co-cultured with pMACs for 24 h without added cytokines, known inducers of pMACs activation. This methodology has advantages over indirect co-culture of TCs and pMACs.

Presently, uterine TCs with typical structure and immunophenotype of double CD-34-positive/vimentin-positive were successfully isolated. When TCs was directly co-cultured with pMACs, an obvious activation/immune response was observed morphologically for TCs-educated pMACs, together with active energy metabolism and an enhanced invasion ability. Furthermore, we found that TCs has the potential to reduce the apoptosis of co-cultured pMACs, together with reduced depletion of ∆Ψm in pMACs. This indicated that TCs-educated pMACs was activated and their apoptosis was inhibited via mitochondrial signaling pathway, leading to an enhanced immune response. We think that TCs–educated macrophages provide an efficient pathway of pMACs activation, and this has an evolutionary advantage since it was activated only by TCs, not by other exogenous agents, such as LPS, etc.

As we know, mitochondria play a crucial role in cell apoptosis or death, loss or depletion of ∆Ψm is a key step in cell apoptosis and leads to the release of diverse pro-apoptotic factors from the mitochondria into the cytoplasm, thus is fatal to cellular activities [55]. Various intrinsic and extrinsic apoptotic pathways can converge at the mitochondrial level and initiated apoptosis, through mitochondrial membrane depolarization [56]. Among these pathways, voltage-dependent potassium channels in mitochondrial, which contribute to the resting membrane potential, are novel mediators in cell apoptosis or death. Mitochondrial K^+^ channels are necessary for regulating various macrophage functions, such as cellular migration, proliferation, activation, and etc. [57]. In addition, an alternation of ∆Ψm can initiate oxidative stress and reactive oxygen species production, which plays an important role and in turn activates its downstream different signaling molecules, ultimately converge into both mitochondria-dependent and mitochondria-independent pathways of apoptosis [58]. In our experiments, based on the observed stabilization of ∆Ψm, we presumed that mitochondrial K+ channels and oxidative stress might be involved in the underlying signaling pathway, by which TCs inhibited apoptosis in pMACs. However, the blind alleys of these fields remain to be explored.

On the other hand, in the current study, direct physical cell-to-cell interaction can promote the development of heterocellular junctions between TCs and pMACs. In a sense, besides paracrine substances in the direct co-cultured system, such type of heterocellular junctions might serve a physiological function of immune synapse, play a pivotal role and was presumably responsible for the observed activation, reduced apoptosis and enhanced invasion of pMACs. However, in our previous in vitro study, in which immune response of pMACs triggered and maintained by TCM in the indirect co-culture system can only be attributable to indirect paracrine effects [38]. Current results further confirm that TCs can be functional players in the initiation and regulation of immune responses of pMACs. Moreover, TCs-educated pMACs might play an important role in EMs and related reproductive physiology.

EMs is a multifactorial disease characterized by the presence of vascularized endometrial tissue at ectopic sites outside the uterine cavity. EMs often results in chronic pelvic pain, dysmenorrhea, dyspareunia, and infertility [44, 59, 60]. Although the exact causes are still poorly understood, increasing studies suggest that, defective immunosurveillance, especially immune dysfunction of pMACs within pelvic microenvironment, plays an important role in the development of EMs [61, 62].

pMACs are one of the important immunocytes in the peritoneal cavity. Generally, in healthy women, pMACs function normally to remove, degrade or engulf tissue debris, such as ectopic endometrium stromal cells and extracellular matrix components of the retrograde menstrual blood in the pelvis, and then reduce the risk of development and/or progression of EMs. While in EMs sites, pMACs were found to be changed significantly, their specific functions differed in early or late stage of EMs. Specifically, in early stage of EMs, pMACs with classical activation (M1) are increasingly recruited to the microenvironment of EMs site. This initial phases of pMACs are considered the major effectors cells of host defense system, playing an indispensable role to recognize, remove or clearance of endogenous ectopic endometrial cellular debris. While in late stage of EMs, the recruited pMACs infiltrates into the endometriotic lesions, where they undergo alternative activation (i.e. M2) [49, 63]. This phases of pMACs were characterized with ineffective recognizing and poor or impaired phagocytic ability to remove viable endometrial cells within peritoneal cavity [45, 64]. pMACs with such phenotype (M2) was involved in homeostasis, tissue repair and remodeling, in turn created an immune tolerant environment, facilitated endometrial cell survival, promoted angiogenesis, attachment, growth and the aggression of ectopic endometrial tissue, etc. [45, 65, 66].

The obtained results were consistent with our previous in vitro evidence, in which pMACs can be activated by TCM [38]. Both of the in vitro studies strengthen the proposed immunoregulatory/immunosurveillance roles of TCs, further indicating that TCs and TCs-educated macrophages potentially play important roles in EMs. Nevertheless, the exact roles or functions of TCs-educated activated pMACs in pathogenesis and pathophysiology of EMs, still need further investigation. For example, their exact roles in the early and late stages of EMs, or the possibility that they facilitate or inhibit the development of EMs, will be promising and attractive topics.

As we know, current therapeutic strategies for EMs are mainly surgery and hormone drugs, with many unavoidable side-effects. On the other hand, TCs transplantation (or together with stem cells) have been shown to be potentially efficacious for treatment of acute myocardial infarction [31, 36]. Among the proposed diverse functions of TCs, most common are the roles of maintaining tissue homeostasis and repairing/remodeling, intercellular communication and immune regulation. Therefore, unique immunological properties of TCs caused great enthusiasm about the potential application for treatment of EMs. It can be proposed that, appropriate modification of TCs and TCs-educated pMACs might be a promising way to restore the defective immunosurveillance of pMACs, led to the enhanced efficacy of medical treatment of EMs.

## Conclusions

Taken together, these data demonstrate that, direct physical cell-to-cell interaction facilitates the formation of heterocellular junctions between TCs and pMACs, presumably responsible for the observed activation of pMACs via mitochondrial signaling pathway. However, a possible involvement of mitochondrial K+ channels, the role of oxidative stress in the signaling pathway of apoptosis in pMACs, and the complex cross-talks among these apoptotic pathways, are still to be elucidated.

Nevertheless, this in vitro study in turn opens an interesting field of TCs biological behavior. TCs-educated pMACs are important mediators in the local immune microenvironment and will be a key factor in the pathological process of EMs. Our findings not only propose the possibility of a new efficient way of pMACs activation, but also provide a promising application of TCs-educated pMACs for treatment of EMs in a simple and clinically feasible fashion.

## Abbreviations

IS: immunological synapse
SEM: scanning electron microscopy
TCM: TC-conditioned media
DXM: dexamethasone
EMs: endometriosis
MFI: mean fluorescence intensity
TCs: telocytes
Tps: telopodes
PI: propidium iodide
pMACs: peritoneal macrophages
UV: ultraviolet
ΔΨm: mitochondrial membrane potential
